# The INFLUENCE 3.0 model: Updated predictions of locoregional recurrence and contralateral breast cancer, now also suitable for patients treated with neoadjuvant systemic therapy

**DOI:** 10.1016/j.breast.2024.103829

**Published:** 2024-10-28

**Authors:** M.C. Van Maaren, T.A. Hueting, D.J.P. van Uden, M. van Hezewijk, L. de Munck, M.A.M. Mureau, P.A. Seegers, Q.J.M. Voorham, M.K. Schmidt, G.S. Sonke, C.G.M. Groothuis-Oudshoorn, S. Siesling, NABOR project group

**Affiliations:** a Department of Health Technology and Services Research, Technical Medical Centre, University of Twente, Enschede, the Netherlands; b Department of Research and Development, Netherlands Comprehensive Cancer Organisation (IKNL), Utrecht, the Netherlands; c Evidencio Medical Decision Support, Haaksbergen, the Netherlands; d Department of Surgical Oncology, Canisius Wilhelmina Hospital, Nijmegen, the Netherlands; e Radiotherapiegroep, Institution for Radiation Oncology, Arnhem, the Netherlands; f Department of Plastic and Reconstructive Surgery, Erasmus MC Cancer Institute, University Medical Center Rotterdam, Rotterdam, the Netherlands; g Palga Foundation, Houten, the Netherlands; h Division of Molecular Pathology, Netherlands Cancer Institute-Antoni van Leeuwenhoek, Amsterdam, the Netherlands; i Department of Medical Oncology, Netherlands Cancer Institute-Antoni van Leeuwenhoek, Amsterdam, the Netherlands

**Keywords:** Breast cancer, Follow-up, Surveillance, Prediction, Locoregional recurrence, Contralateral breast cancer

## Abstract

**Background:**

Individual risk prediction of 5-year locoregional recurrence (LRR) and contralateral breast cancer (CBC) supports decisions regarding personalised surveillance. The previously developed INFLUENCE tool was rebuild, including a recent population and patients who received neoadjuvant systemic therapy (NST).

**Methods:**

Women, surgically treated for nonmetastatic breast cancer, diagnosed between 2012 and 2016, were selected from the Netherlands Cancer Registry. Cox regression with restricted cubic splines was compared to Random Survival Forest (RSF) to predict five-year LRR and CBC risks. Separate models were developed for NST patients. Discrimination and calibration were assessed by 100x bootstrap resampling.

**Results:**

In the non-NST and NST group, 49,631 and 10,154 patients were included, respectively. Age, mode of detection, histology, sublocalisation, grade, pT, pN, hormonal receptor status ± endocrine treatment, HER2 status ± targeted treatment, surgery ± immediate reconstruction ± radiation therapy, and chemotherapy were significant predictors for LRR and/or CBC in non-NST patients. For NST patients this was similar, but excluding (y)pT and (y)pN status, and including presence of ductal carcinoma in situ, axillary lymph node dissection and pathologic complete response.

For non-NST patients, the Cox and RSF models were integrated in the online tool with 5-year AUCs of 0.77 (95%CI:0.77–0.77) and 0.68 (95%CI:0.67–0.68)] for LRR and CBC prediction, respectively. For NST patients, the RSF model performed best (AUCs 0.77 (95%CI:0.76–0.78) and 0.73 (95%CI:0.69–0.76) for LRR and CBC, respectively). Regarding calibration, observed-predicted differences were all <1 %.

**Conclusion:**

This INFLUENCE 3.0 models showed moderate performance in LRR and CBC prediction. The models have been made available as online tool to enable clinical decision support regarding personalised follow-up.

## Highlights

• INFLUENCE 3.0 moderately predicts breast cancer recurrence after curative treatment.
• INFLUENCE 3.0 can support decision-making regarding frequency of surveillance.
• INFLUENCE 3.0 contributes to personalised follow-up in breast cancer patients.
• INFLUENCE 3.0 is available on Evidencio, a platform for medical prediction models.

## 1 Introduction

In 2023, over 15,000 women were diagnosed with invasive breast cancer in the Netherlands [1]. While breast cancer incidence has increased, mortality rates have declined [2]. Yearly, many breast cancer survivors enter the surveillance program as part of follow-up care, aiming to early detect locoregional recurrences (LRR) or second primary contralateral breast cancers (CBC) [3]. The Dutch breast cancer guideline recommends annual mammography or MRI (depending on risk category) combined with physical examination for five years [3]. This schedule is not tailored to LRR and CBC risks, which are largely influenced by individual patient-, tumour- and treatment-related characteristics [4]. This, alongside the low overall LRR and CBC risks [5], likely results in unnecessary visits for many patients, which may cause stress [6,7] and increased burden to the healthcare system [8].

Hence, the INFLUENCE 1.0 model was developed [1] and later updated to INFLUENCE 2.0(4). INFLUENCE 2.0 estimates, amongst others, time-dependent risks of LRR and CBC within five years following surgery. This model was designed for personalised decision support regarding scheduling of surveillance visits. However, neoadjuvant systemic therapy (NST) was not applied to a sufficient degree during the timeframe of the included cohort, and thus, INFLUENCE 2.0 was not suitable for patients treated with NST. Furthermore, the INFLUENCE 2.0 model was based on data from patients diagnosed in 2007, 2008 or the first quarter of 2012. To reflect current clinical practice in which NST is increasingly applied [9,10], we developed and validated a new INFLUENCE 3.0 model including a more recent population and including patients treated with NST.

## 2 Methods

### 2.1 Study design and population

Data on women, surgically treated for primary invasive nonmetastatic breast cancer, diagnosed between 2012 and 2016, were derived from the Netherlands Cancer Registry (NCR). The NCR is a population-based registry containing all newly diagnosed malignancies (including subsequent primary tumours) from 1989 on. Trained registrars prospectively collect data on patient-, tumour- and treatment-related characteristics directly from patient files. Vital status and date of death (if applicable) were retrieved through linkage with the municipal personal records database (last linkage February 2022).

Patients with synchronous breast cancer (second primary tumour within 90 days) or treated with surgery in a foreign hospital were excluded. Additional exclusion criteria were male gender, incidental findings, positive tumour margins after surgery, unknown surgery date or unavailable follow-up. The population was divided in two separate cohorts. The non-NST group consisted of patients treated without systemic therapy or adjuvant systemic therapy. The NST group consisted of patients treated with either neoadjuvant chemotherapy, endocrine therapy, or both. In the non-NST group, patients with pT0, unknown pT or pN were additionally excluded. In the NST group, patients with cT0, cTIS, unknown cT or cN were excluded, as well as patients who only received neoadjuvant radiation therapy or targeted therapy without chemotherapy.

### 2.2 Additional data collection

For INFLUENCE 1.0 and 2.0, data on LRRs was collected manually by going back to patient files of all patients diagnosed in specific cohorts [1,4], which is a time-consuming way of data gathering. Here, a linkage with the Dutch Nationwide Pathology Databank (Palga) [11] was executed. Based on an algorithm including date of diagnosis and tumour topography, patients suspected of having had a LRR were selected. Consequently, registrars of the NCR gathered data on these LRRs from patient files, if applicable. Patients not suspected to have had a LRR were assumed to be LRR-free. This way of data collection was validated by analysing all LRRs of patients diagnosed in the first quarter of 2012. For this specific cohort, we earlier performed manual data collection on LRRs, and could thus serve as a gold standard. Based on this validation, the number of LRRs was estimated to be ±80 % complete.

### 2.3 Outcomes and definitions

The primary outcomes were LRR and CBC, which were defined as reappearance of breast cancer in ipsilateral breast tissue or regional nodes (LRR) or the contralateral breast (CBC) [12]. Follow-up was defined as the time between date of definite surgery and event or last observation. In case of multiple events, only the first event was considered. Correction for competing risks was performed by censoring patients at the date another event (distant metastasis or death) occurred [13]. Pathological complete response (pCR) was defined as ypT0N0 or ypTISN0.

### 2.4 Model development and validation

Missing data were considered to be missing at random, and were subsequently imputed five times using the mice package in R (default settings). Estimates of imputed datasets were pooled and compared with estimates obtained from complete case analysis. We compared the performance of multivariable Cox proportional hazard models with that of random survival forest (RSF) models, for the non-NST and NST group separately. Cox models are most frequently used for time-to-event data, but compared to RSF models they are less flexible and limited to interaction effects that are manually included in the model. RSF considers all possible relationships between variables [14]. In the Cox model, restricted cubic splines with three knots were fitted to account for the potential non-linear effect of age [15]. Knots were put on the 10th, 50th and 90th percentile of the distribution, which equalled ages 46, 62 and 76.3 in the dataset. The proportional hazard assumption was tested by visually inspecting Schoenfeld residuals over time [16]. For RSF, the rfsrc function in R was used with the following parameters: splitrule = "bs.gradient", mtry = 5, ntree = 200, nodedepth = 5, nodesize = 15. Variables were included based on clinical foreknowledge and univariable analysis using Cox models. Manual backward selection was performed to remove variables not significantly contributing to the multivariable model (using Likelihood Ratio tests). Information on surgery, immediate breast reconstruction and radiation therapy was combined into one variable to avoid collinearity. The same was done for receptor statuses and accompanying treatment, with one exception: HER2 status was in the NST cohort not combined with targeted therapy, as 99.4 % of the HER2 positive patients received targeted therapy in this group, resulting in too little events in the category without targeted therapy to be modeled. For the Cox model, variable importance was illustrated by hazard ratios (HRs) and 95 % confidence intervals (CIs). For the RSF models, the importance of variables relative to each other was analysed [17]. Calibration was expressed as the Integrated Calibration Index [18] (ICI, weighted difference between observed and predicted probabilities), the E50 (median absolute difference between observed and predicted probabilities) and E90 (90th percentile of the absolute difference). The closer to zero, the better the calibration [18]. In case of ICI values < 0.01 or < -0.01, a model was considered to be well calibrated. Discrimination was expressed as the area under the receiver operating characteristic curve (AUC) at quarterly time intervals. Values between 0.5 and 0.7, 0.7–0.8 and ≥ 0.8 were defined as poor, moderate and good performance, respectively [19].

Models were developed and validated using 100 bootstrap samples (equal size as population size, with replacement). Discrimination and calibration were analysed in the bootstrap sample used to develop the models (original performance) and in the full dataset (true performance). The difference between original and true performance is the model's optimism (a measure for overfitting). By subtracting the optimism from the true performance, the (optimism-corrected) adjusted performance was obtained [20,21].

The statistical software program R, version 4.2.2 was used for all analyses. The best-performing models were integrated in an online tool on https://www.evidencio.com/models/show/2238.

## 3 Results

**Fig. 1:**
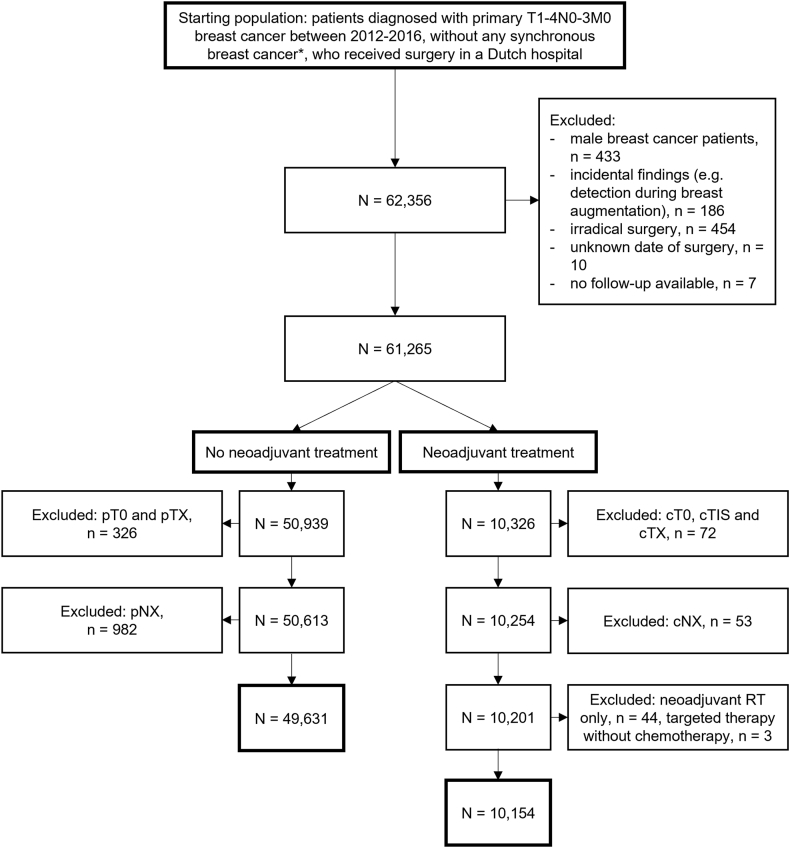
Flowchart of patient selection. ∗a second primary breast tumour diagnosed within 90 days of the first.

Finally, 49,631 patients were included in the non-NST group and 10,154 patients in the NST group (Fig. 1). Baseline characteristics of both cohorts are shown in Table 1. In the non-NST group, 1090 (2.2 %) and 1566 (3.2 %) patients experienced a LRR or CBC as first event, respectively, during a median follow-up of 7.7 years (IQR:6.4–9.0 years). In the NST group, 334 (3.3 %) and 199 (2.0 %) patients experienced a LRR or CBC as first event during a median follow-up of 6.6 years (IQR:5.5–7.9 years).

**Table 1:** Baseline characteristics of the two cohorts: patients treated without neoadjuvant systemic treatment (no systemic therapy or adjuvant systemic therapy only), and patients treated with neoadjuvant systemic treatment.

	No neoadjuvant systemic treatment (no systemic treatment or only adjuvant) (non-NST) (N = 49,631)	Neoadjuvant systemic treatment (NST) (N = 10,154)
**Year of diagnosis**		
2012	10,593 (21.3 %)	1341 (13.2 %)
2013	10,243 (20.6 %)	1688 (16.6 %)
2014	10,023 (20.2 %)	2024 (19.9 %)
2015	9413 (19.0 %)	2575 (25.4 %)
2016	9359 (18.9 %)	2526 (24.9 %)
**Age (years)**		
Median (IQR)	62 (52–70)	50 (44–60)
<40	1660 (3.3 %)	1424 (14.0 %)
40-49	6837 (13.8 %)	3269 (32.2 %)
50-59	13,021 (26.2 %)	2737 (27.0 %)
60-69	15,324 (30.9 %)	1889 (18.6 %)
70–79	9440 (19.0 %)	547 (5.4 %)
>79	3349 (6.7 %)	288 (2.8 %)
**Menopausal status**		
premenopausal	7731 (15.6 %)	4003 (39.4 %)
perimenopausal	2354 (4.7 %)	673 (6.6 %)
postmenopausal	34,958 (70.4 %)	4264 (42.0 %)
unknown	4588 (9.2 %)	1214 (12.0 %)
**Socioeconomic status∗**		
low	14,531 (29.3 %)	2651 (26.1 %)
medium	19,657 (39.6 %)	3929 (38.7 %)
high	15,443 (31.1 %)	3574 (35.2 %)
**Mode of detection**		
Clinically detected	25,747 (51.9 %)	8352 (82.3 %)
Screen-detected	23,355 (47.1 %)	1544 (15.2 %)
unknown	529 (1.1 %)	258 (2.5 %)
**Laterality**		
left	25,212 (50.8 %)	5276 (52.0 %)
right	24,416 (49.2 %)	4878 (48.0 %)
unknown	3 (0.0 %)	–
**Sublocalisation**		
outer quadrants (C50.4–6)	23,515 (47.4 %)	4524 (44.6 %)
inner quadrants (C50.2–3)	10,118 (20.4 %)	1635 (16.1 %)
central parts (C50.0–1)	3858 (7.8 %)	748 (7.4 %)
overlapping lesions (C50.8)	11,474 (23.1 %)	3139 (30.9 %)
unknown	666 (1.3 %)	108 (1.1 %)
**Histological tumour type**		
ductal	40,321 (81.2 %)	8609 (84.8 %)
lobular	5570 (11.2 %)	1046 (10.3 %)
mixed ductal lobular	1425 (2.9 %)	233 (2.3 %)
other	2315 (4.7 %)	266 (2.6 %)
**Differentiation grade**		
grade 1	12,865 (25.9 %)	790 (7.8 %)
grade 2	22,829 (46.0 %)	3102 (30.5 %)
grade 3	12,449 (25.1 %)	2378 (23.4 %)
unknown	1488 (3.0 %)	3884 (38.3 %)
**Multifocality**		
no	42,343 (85.3 %)	7312 (72.0 %)
yes	7112 (14.3 %)	2727 (26.9 %)
unknown	176 (0.4 %)	115 (1.1 %)
**Clinical tumour stage**		
cTIS	1119 (2.3 %)	–
cT1	32,918 (66.3 %)	1553 (15.3 %)
cT2	12,898 (26.0 %)	5870 (57.8 %)
cT3	1195 (2.4 %)	1959 (19.3 %)
cT4	253 (0.5 %)	772 (7.6 %)
unknown	1248 (2.5 %)	**-**
**Clinical nodal stage**		
cN0	44,726 (90.1 %)	4226 (41.6 %)
cN1	4401 (8.9 %)	5024 (49.5 %)
cN2	65 (0.1 %)	203 (2.0 %)
cN3	68 (0.1 %)	701 (6.9 %)
unknown	371 (0.7 %)	–
**Pathological tumour classification**		
pT0	–	2358 (23.2 %)
pTIS	–	410 (4.0 %)
pT1	34,677 (69.9 %)	4052 (39.9 %)
pT2	13,502 (27.2 %)	2351 (23.2 %)
pT3	1213 (2.4 %)	632 (6.2 %)
pT4	239 (0.5 %)	116 (1.1 %)
unknown	–	235 (2.3 %)
**Pathological nodal classification**		
pN0	35,116 (70.8 %)	5284 (52.0 %)
pN1	11,944 (24.1 %)	3234 (31.8 %)
pN2	1631 (3.3 %)	818 (8.1 %)
pN3	940 (1.9 %)	372 (3.7 %)
unknown	–	446 (4.4 %)
**Hormonal receptor status + treatment**		
positive∗∗ + endocrine therapy	26,610 (53.6 %)	6823 (67.2 %)
positive∗∗ – endocrine therapy	16,270 (32.8 %)	364 (3.6 %)
negative	6267 (12.6 %)	2858 (28.1 %)
unknown	484 (1.0 %)	109 (1.1 %)
**HER2 status + treatment**		
negative	43,032 (86.7 %)	7438 (73.3 %)
positive + targeted therapy	3665 (7.4 %)	2445 (24.1 %)
positive – targeted therapy	1581 (3.2 %)	66 (0.7 %)
unknown∗∗∗	1353 (2.7 %)	205 (2.0 %)
**Ductal carcinoma in situ present**		
no	23,843 (48.0 %)	6214 (61.2 %)
yes	25,167 (50.7 %)	3612 (35.6 %)
unknown	621 (1.3 %)	328 (3.2 %)
**Molecular diagnostics∗∗∗∗**		
no	44,630 (89.9 %)	9903 (97.5 %)
yes, low risk	2990 (6.0 %)	97 (1.0 %)
yes, high risk	1767 (3.6 %)	142 (1.4 %)
yes, unknown result	244 (0.5 %)	12 (0.1 %)
**Type of surgery + RT + immediate breast reconstruction**		
BCS + RT	31,692 (63.9 %)	4947 (48.7 %)
BCS – RT	689 (1.4 %)	153 (1.5 %)
mastectomy + RT – immediate breast reconstruction	4008 (8.1 %)	2718 (26.8 %)
mastectomy + RT + immediate breast reconstruction	682 (1.4 %)	667 (6.6 %)
mastectomy – RT – immediate breast reconstruction	9420 (19.0 %)	876 (8.6 %)
mastectomy – RT + immediate breast reconstruction	3140 (6.3 %)	793 (7.8 %)
**Axillary lymph node dissection**		
no	42,072 (84.8 %)	6454 (63.6 %)
yes	7559 (15.2 %)	3700 (36.4 %)
**Chemotherapy**		
no	33,078 (66.6 %)	863 (8.5 %)
yes	16,553 (33.4 %)	9291 (91.5 %)
**Pathologic complete response**		
no	NA	7527 (74.1 %)
yes	NA	2215 (21.8 %)
unknown	NA	412 (4.1 %)
**Vital status at end of follow-up**		
alive	42,007 (84.6 %)	8385 (82.6 %)
deceased (all causes)	7624 (15.4 %)	1769 (17.4 %)
**Locoregional recurrence as first event**		
no	48,541 (97.8 %)	9820 (96.7 %)
yes	1090 (2.2 %)	334 (3.3 %)
**Contralateral breast tumour as first event**		
no	48,065 (96.8 %)	9955 (98.0 %)
yes	1566 (3.2 %)	199 (2.0 %)

∗SES was based on scores assigned to the four numbers of the Dutch postal code, extracted from the Netherlands Institute for Social Research.∗∗either ER or PR positive or both. ∗∗∗includes 2+ result of immunohistochemistry (0.3 %). ∗∗∗∗either oncotype DX or mammaprint. intermediate risk oncotype (<0.1 %) classified as low risk.

Abbreviations: NST = neoadjuvant systemic treatment, IQR = interquartile range, p = pathological (post-operative), T = tumour, N = nodal, IS = in situ, HER2 = human epidermal growth factor receptor 2, NA = not applicable, BCS = breast-conserving surgery, RT = radiation therapy.

### 3.1 Non-NST

**Fig. 2:**
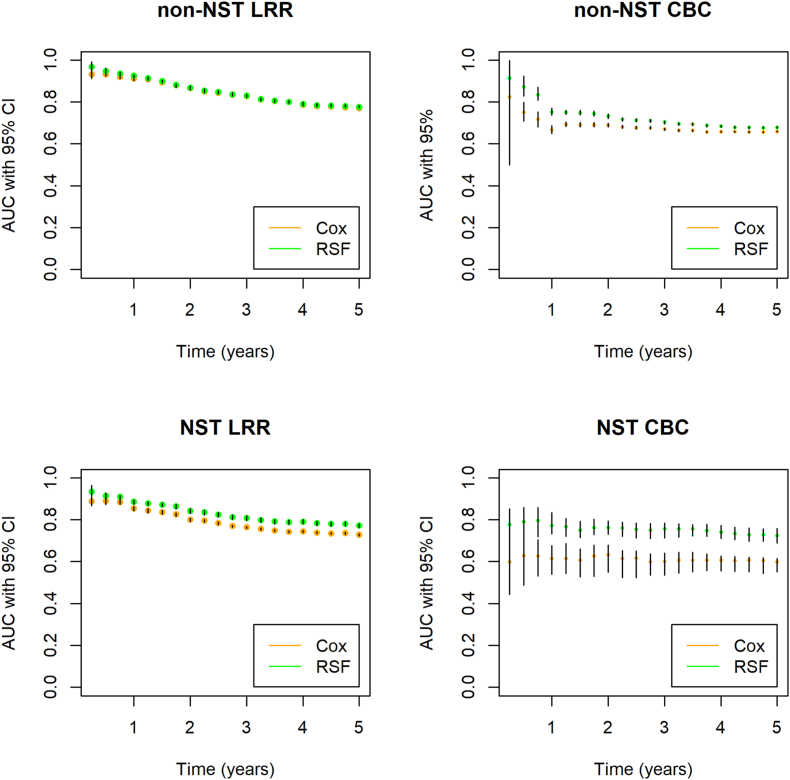
**Mean hazard ratios and 95 % confidence intervals of 100 bootstrap samples on which the Cox regression model predicting LRR in the non-NST cohort is developed.** ∗ As age is modeled using restricted cubic splines, the interpretation of the coefficients is not straightforward, as the effect of age on risk of LRR is a function of multiple regression coefficients. Abbreviations: LRR = locoregional recurrence, NST = neoadjuvant systemic treatment, HR = hazard ratio, LCL = lower confidence limit, UCL = upper confidence limit, pT = pathological tumour classification, pN = pathological nodal classification, HER2 = human epidermal growth factor 2, RT = radiation therapy.

**Fig. 3:**
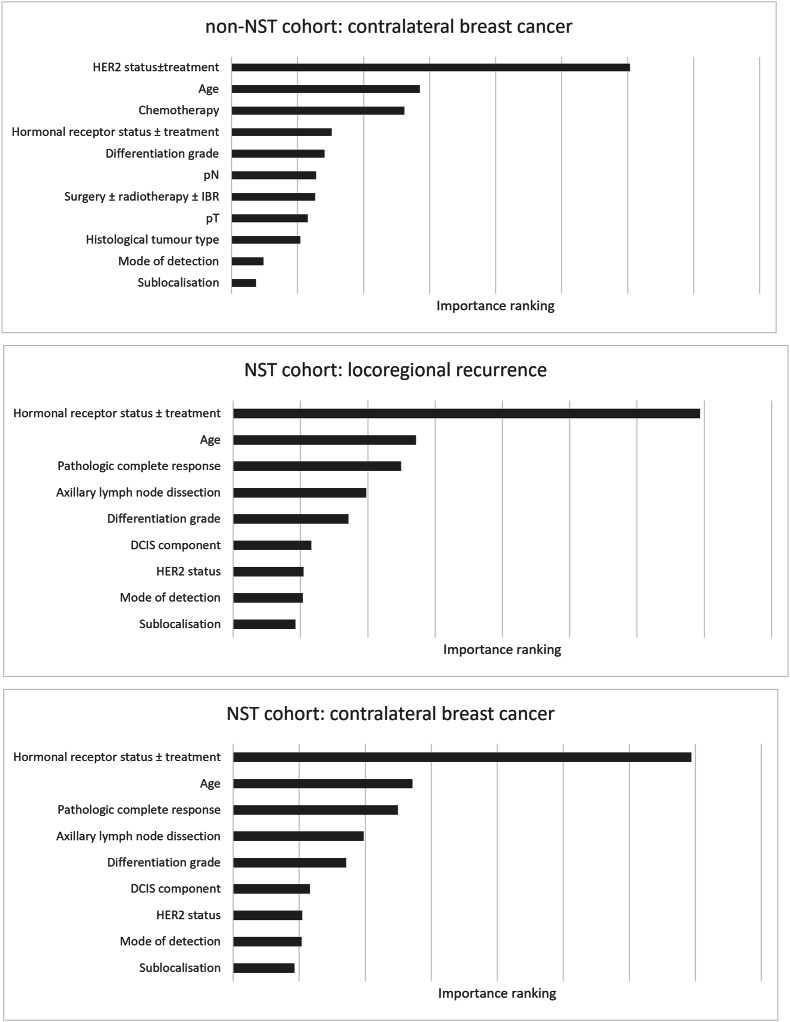
**For optimism corrected area under the curves for the non-NST and NST cohorts separately.** The left panels show AUCs for prediction of LRR, using the Cox and RSF models. The right panels show AUCs for prediction of CBC, using the Cox and RSF models. Abbreviations: NST = neoadjuvant systemic treatment, LRR = locoregional recurrence, CBC = contralateral breast cancer, AUC = area under the receiver operating characteristic curve, CI = confidence interval.

Predictors included in the final models were age (as continuous variable), mode of detection (screening through the national programme or clinically detected), histology, tumour sublocalisation, grade, pT, pN, hormonal receptor status ± endocrine treatment, HER2 status ± targeted treatment, surgery ± immediate breast reconstruction ± radiation therapy, and chemotherapy. No violations of the Cox proportional hazards assumption were found. The importance of variables in the Cox model for LRR is visualized in Fig. 2 and presented in Supplementary Table 2. Fig. 3 shows variable importances in the RSF model, predicting CBC.

At year 1, optimism-corrected AUCs for LRR prediction were the highest (0.91, 95%CI:0.90–0.92) and 0.93, (95%CI:0.92–0.93) for Cox and RSF, respectively). The AUCs slightly decreased over time (Fig. 3). Five-year AUCs were 0.77 (95%CI:0.77–0.77) and 0.78 (95%CI:0.77–0.78), respectively. Optimism was on average smaller for Cox than for RSF, reflected by a decrease in AUC of 0.008 and 0.023 in Cox and RSF (original 5-year AUCs were 0.78 and 0.80), respectively (Table 2 and Supplementary Table 1 for detailed values per quarterly time interval). For CBC prediction, AUCs were more constant over time (Fig. 3). Five-year AUCs for prediction of CBC were 0.66 (95%CI:0.66–0.66) and 0.68 (95%CI:0.67–0.68) for Cox and RSF, respectively. Optimism was 0.011 and 0.028 for Cox and RSF (original AUCs were 0.67 and 0.71), respectively (Table 2, Supplementary Table 1).

**Table 2:** Discrimination results per yearly time interval.

		Time (years)	Cox									RSF								
			Original			Adjusted			Optimism			Original			Adjusted			Optimism		
			LB	AUC	UB	LB	AUC	UB	LB	mean	UB	LB	AUC	UB	LB	AUC	UB	LB	mean	UB
**Non-NST**	**LRR**	1	0.89	**0.92**	0.93	0.90	**0.91**	0.92	−0.014	**0.004**	0.016	0.93	**0.94**	0.96	0.92	**0.93**	0.93	0.014	**0.011**	0.025
		2	0.85	**0.87**	0.88	0.86	**0.87**	0.87	−0.005	**0.002**	0.011	0.87	**0.88**	0.90	0.86	**0.87**	0.88	0.015	**0.016**	0.019
		3	0.82	**0.83**	0.85	0.82	**0.83**	0.83	−0.004	**0.006**	0.017	0.84	**0.85**	0.86	0.82	**0.83**	0.84	0.016	**0.020**	0.026
		4	0.78	**0.79**	0.81	0.78	**0.79**	0.79	−0.002	**0.008**	0.017	0.80	**0.81**	0.83	0.78	**0.79**	0.80	0.018	**0.024**	0.029
		5	0.77	**0.78**	0.79	0.77	**0.77**	0.77	−0.001	**0.008**	0.017	0.79	**0.80**	0.81	0.77	**0.78**	0.78	0.015	**0.023**	0.025
	**CBC**	1	0.62	**0.67**	0.73	0.65	**0.67**	0.69	−0.025	**0.008**	0.043	0.80	**0.83**	0.84	0.74	**0.75**	0.77	0.064	**0.073**	0.069
		2	0.67	**0.70**	0.73	0.68	**0.69**	0.70	−0.011	**0.008**	0.036	0.76	**0.77**	0.79	0.72	**0.73**	0.74	0.039	**0.041**	0.047
		3	0.66	**0.68**	0.70	0.66	**0.67**	0.68	−0.003	**0.008**	0.023	0.73	**0.74**	0.75	0.69	**0.70**	0.71	0.031	**0.035**	0.044
		4	0.65	**0.67**	0.68	0.65	**0.66**	0.66	−0.004	**0.008**	0.021	0.71	**0.72**	0.73	0.68	**0.68**	0.69	0.027	**0.033**	0.042
		5	0.65	**0.67**	0.68	0.66	**0.66**	0.66	−0.001	**0.011**	0.021	0.70	**0.71**	0.72	0.67	**0.68**	0.68	0.024	**0.028**	0.036
**NST**	**LRR**	1	0.81	**0.86**	0.89	0.84	**0.85**	0.86	−0.030	**0.005**	0.030	0.89	**0.91**	0.93	0.87	**0.89**	0.89	0.018	**0.027**	0.034
		2	0.76	**0.81**	0.84	0.79	**0.80**	0.81	−0.032	**0.008**	0.035	0.86	**0.89**	0.90	0.83	**0.84**	0.85	0.031	**0.043**	0.048
		3	0.73	**0.77**	0.81	0.76	**0.76**	0.77	−0.028	**0.009**	0.038	0.84	**0.86**	0.87	0.80	**0.81**	0.82	0.042	**0.049**	0.056
		4	0.72	**0.75**	0.78	0.74	**0.74**	0.75	−0.018	**0.010**	0.034	0.82	**0.84**	0.86	0.78	**0.79**	0.80	0.042	**0.051**	0.061
		5	0.71	**0.74**	0.77	0.72	**0.73**	0.73	−0.013	**0.009**	0.038	0.81	**0.83**	0.84	0.76	**0.77**	0.78	0.044	**0.053**	0.063
	**CBC**	1	0.56	**0.65**	0.76	0.54	**0.62**	0.68	0.022	**0.037**	0.082	0.89	**0.91**	0.94	0.73	**0.77**	0.83	0.159	**0.144**	0.111
		2	0.61	**0.67**	0.73	0.55	**0.63**	0.68	0.063	**0.036**	0.057	0.86	**0.89**	0.92	0.73	**0.76**	0.79	0.131	**0.127**	0.121
		3	0.61	**0.63**	0.71	0.53	**0.60**	0.64	0.072	**0.031**	0.070	0.85	**0.87**	0.90	0.71	**0.76**	0.78	0.135	**0.118**	0.118
		4	0.61	**0.65**	0.69	0.55	**0.61**	0.63	0.055	**0.041**	0.062	0.83	**0.86**	0.88	0.71	**0.74**	0.77	0.117	**0.119**	0.106
		5	0.61	**0.64**	0.69	0.55	**0.60**	0.61	0.060	**0.044**	0.080	0.81	**0.84**	0.87	0.69	**0.73**	0.76	0.123	**0.114**	0.107

Abbreviations: LB = 95 % lower bound. AUC = area under the receiver operating characteristic curve. UB = 95 % upper bound. Mean values are depicted in bold. 5-year for optimism adjusted AUCs are shaded.

All models showed good calibration, reflected by ICI values ≤ 0.01 (observed-predicted difference≤1 %) (Table 3).

**Table 3:** Calibration results per yearly time interval.

		Time (years)	Cox									RSF								
			ICI			E50			E90			ICI			E50			E90		
			LB	Mean	UB	LB	Mean	UB	LB	Mean	UB	LB	Mean	UB	LB	Mean	UB	LB	Mean	UB
**Non-NST**	**LRR**	1	0.0001	**0.0002**	0.0004	0.0000	**0.0001**	0.0004	0.0001	**0.0003**	0.0009	0.0002	**0.0003**	0.0005	0.0001	**0.0002**	0.0004	0.0002	**0.0004**	0.0009
		2	0.0002	**0.0003**	0.0008	0.0000	**0.0002**	0.0007	0.0003	**0.0004**	0.0016	0.0015	**0.0020**	0.0023	0.0006	**0.0014**	0.0016	0.0014	**0.0018**	0.0028
		3	0.0003	**0.0005**	0.0009	0.0002	**0.0003**	0.0008	0.0003	**0.0008**	0.0016	0.0033	**0.0040**	0.0043	0.0020	**0.0028**	0.0031	0.0033	**0.0043**	0.0051
		4	0.0003	**0.0008**	0.0012	0.0002	**0.0004**	0.0011	0.0004	**0.0010**	0.0023	0.0053	**0.0059**	0.0064	0.0035	**0.0044**	0.0049	0.0054	**0.0069**	0.0083
		5	0.0004	**0.0008**	0.0015	0.0002	**0.0005**	0.0014	0.0006	**0.0011**	0.0028	0.0072	**0.0080**	0.0086	0.0048	**0.0058**	0.0066	0.0075	**0.0092**	0.0107
	**CBC**	1	0.0001	**0.0002**	0.0005	0.0001	**0.0001**	0.0004	0.0002	**0.0003**	0.0009	0.0001	**0.0003**	0.0005	0.0001	**0.0002**	0.0006	0.0003	**0.0005**	0.0011
		2	0.0002	**0.0004**	0.0008	0.0002	**0.0004**	0.0008	0.0003	**0.0007**	0.0017	0.0006	**0.0010**	0.0014	0.0002	**0.0008**	0.0012	0.0008	**0.0019**	0.0032
		3	0.0003	**0.0007**	0.0013	0.0003	**0.0006**	0.0011	0.0006	**0.0012**	0.0026	0.0014	**0.0025**	0.0031	0.0013	**0.0020**	0.0027	0.0021	**0.0048**	0.0066
		4	0.0003	**0.0009**	0.0017	0.0002	**0.0008**	0.0016	0.0006	**0.0015**	0.0032	0.0032	**0.0043**	0.0049	0.0027	**0.0038**	0.0043	0.0049	**0.0083**	0.0104
		5	0.0004	**0.0011**	0.0021	0.0003	**0.0010**	0.0020	0.0007	**0.0019**	0.0039	0.0043	**0.0056**	0.0064	0.0038	**0.0048**	0.0056	0.0069	**0.0109**	0.0145
**NST**	**LRR**	1	0.0010	**0.0015**	0.0024	0.0007	**0.0013**	0.0019	0.0015	**0.0024**	0.0057	0.0007	**0.0014**	0.0026	0.0004	**0.0010**	0.0022	0.0011	**0.0021**	0.0047
		2	0.0017	**0.0025**	0.0038	0.0011	**0.0021**	0.0030	0.0029	**0.0039**	0.0088	0.0014	**0.0035**	0.0052	0.0005	**0.0021**	0.0039	0.0015	**0.0038**	0.0086
		3	0.0022	**0.0031**	0.0049	0.0014	**0.0027**	0.0040	0.0035	**0.0051**	0.0109	0.0029	**0.0060**	0.0083	0.0012	**0.0038**	0.0063	0.0033	**0.0075**	0.0157
		4	0.0026	**0.0037**	0.0054	0.0017	**0.0033**	0.0048	0.0039	**0.0060**	0.0117	0.0051	**0.0085**	0.0116	0.0026	**0.0056**	0.0088	0.0061	**0.0116**	0.0203
		5	0.0028	**0.0040**	0.0062	0.0019	**0.0035**	0.0052	0.0042	**0.0065**	0.0126	0.0069	**0.0106**	0.0140	0.0035	**0.0069**	0.0109	0.0085	**0.0152**	0.0233
	**CBC**	1	0.0004	**0.0007**	0.0018	0.0002	**0.0005**	0.0020	0.0007	**0.0013**	0.0026	0.0004	**0.0006**	0.0017	0.0003	**0.0005**	0.0015	0.0006	**0.0013**	0.0027
		2	0.0006	**0.0012**	0.0020	0.0004	**0.0010**	0.0021	0.0011	**0.0020**	0.0045	0.0004	**0.0012**	0.0019	0.0002	**0.0009**	0.0019	0.0006	**0.0016**	0.0036
		3	0.0007	**0.0015**	0.0027	0.0006	**0.0013**	0.0025	0.0012	**0.0027**	0.0055	0.0013	**0.0020**	0.0032	0.0005	**0.0014**	0.0024	0.0015	**0.0021**	0.0056
		4	0.0010	**0.0020**	0.0035	0.0007	**0.0018**	0.0034	0.0019	**0.0039**	0.0071	0.0025	**0.0039**	0.0054	0.0009	**0.0027**	0.0043	0.0028	**0.0044**	0.0082
		5	0.0012	**0.0025**	0.0043	0.0011	**0.0022**	0.0040	0.0021	**0.0045**	0.0086	0.0030	**0.0047**	0.0063	0.0015	**0.0036**	0.0053	0.0038	**0.0054**	0.0090

Values presented are for optimism adjusted values. Mean values are depicted in bold. Abbreviations: Cox = multivariable Cox model with restricted cubic splines. RSF = random survival forest model. NST = neoadjuvant systemic treatment. LRR = locoregional recurrence. CBC = contralateral breast cancer. ICI = integrated calibration index. E50 = the median absolute difference between observed and expected predictions. E90 = the 90th percentile of the absolute difference between observed and expected predictions. LB = 95 % lower bound. UB = 95 % upper bound.

The Cox and RSF models were integrated in the online tool for prediction of LRR and CBC, respectively.

### 3.2 NST

Predictors included in the final models were age, mode of detection, sublocalisation, presence of ductal carcinoma in situ (DCIS), differentiation grade, hormonal receptor status ± endocrine treatment, HER2 status, axillary lymph node dissection and pCR. The importance of variables in the models are shown in Supplementary Fig. 1.

At year 1, AUCs for prediction of LRR were the highest (0.85, 95%CI:0.84–0.86) and 0.89 (95%CI:0.87–0.89), for the Cox and RSF models, respectively. Over time, the AUCs slightly decreased (Fig. 3). The 5-year AUCs were 0.73 (95%CI:0.72–0.73) and 0.77 (95%CI:0.76–0.78), respectively (Table 2, Supplementary Table 1).

For prediction of CBC, AUCs remained more constant (Fig. 3). Five-year AUCs for prediction of CBC were 0.60 (95%CI:0.55–0.61) and 0.73 (95%CI:0.69–0.76) for Cox and RSF, respectively.

Optimism was on average higher for RSF than for Cox, for both outcomes (Table 2, Supplementary Table 2).

Both models show good calibration in LRR and CBC prediction, reflected by ICI, E50 and E90 values < 0.01 (observed-predicted difference <1 %) (Table 3).

Based on these observations the RSF models were integrated in the online tool for prediction of both LRR and CBC.

For all models, estimates obtained with imputed data were similar to complete case analyses.

## 4 Discussion

INFLUENCE 3.0 models were designed to support the shared decision-making process regarding personalised surveillance after curative treatment. INFLUENCE 3.0 is explicitly not meant to be used for treatment decision-making, because information on treatment was collected retrospectively, meaning that treatment allocation was not random [22].

For the non-NST cohort, both Cox and RSF models performed similar in LRR risk prediction. The Cox model was incorporated in the online tool to predict LRR, as it showed less optimism in prediction, which increases its potential accuracy in an external population [20]. Besides, Cox models are better interpretable than RSF models. The RSF model predicted CBC best. In the NST cohort, RSF performed best in both prediction of LRR and CBC.

Calibration of all models was good. Discrimination of the models predicting LRR was moderate with optimism-corrected 5-year AUCs of 0.77 (95%CI:0.77–0.77) and 0.77 (95%CI:0.76–0.78) for the non-NST (Cox) and NST cohort (RSF), respectively. The models predicting CBC showed poor to moderate discrimination, with optimism-corrected AUCs of 0.68 (95%CI:0.67–0.69) and 0.73 (95%CI:0.69–0.76) for the non-NST and NST cohort, respectively. In the NST cohort there was a higher degree of optimism for the RSF model predicting CBC, compared to the Cox model. This may have been caused by the relatively lower numbers of events in this population. A high degree of optimism is an indicator of overfitting, meaning that the true performance of the model may be lower [20]. This is more likely to occur in machine learning-based models, as these are able to incorporate complex variable relationships that are specific for a certain dataset. This may lead to a lower performance in another population [23]. We used bootstrap resampling to obtain optimism-adjusted performance measures, and the results still indicated moderate performance of the RSF model – which was much better than the Cox model. As INFLUENCE will initially be used in the Dutch breast cancer population, and because the coefficients were also corrected for optimism, we are confident that the model provides valid results in the Netherlands. External validation should be performed to ensure validity of the model in other populations.

Here, we labelled AUC values of 0.68–0.77 as poor to moderate. Still, we recommend to use the INFLUENCE 3.0 model in clinical practice. The reason for this is that AUC labelling is arbitrary (in many papers an AUC of 0.68 is considered to be moderate and an AUC of 0.77 as good) [19], and we would like readers to look beyond AUC values only. Performance of a prediction model should not be judged based on AUCs alone. Calibration of risk predictions is of utmost importance in predictive modelling. Even with high AUCs, calibration can be poor, resulting in incorrect risk estimates and consequently harmful clinical decisions [24]. After validation, the INFLUENCE 3.0 model showed good calibration. In combination with these AUCs, this supports the clinical validity of the INFLUENCE 3.0 model and justifies further evaluation of its clinical utility for (non-hereditary) patients.

The declining AUC over time in LRR prediction could possibly be attributed to the typical LRR pattern over time. LRR risks are highest in years 2–3 and decline thereafter [25]. It is plausible that a model can better discriminate between patients with and without LRR in the first years due to this typical pattern.

The lower performance of the models predicting CBC may be caused by the fact that unmeasured factors such as genetic predisposition and family history – which are known key factors associated with CBC [26] – could not be included in the model. However, a large study in which a model predicting CBC risk was developed (PREDICTCBC-2.0) showed that, even in the presence of information on family history, BMI and important gene mutations, the model was only able to moderately discriminate between cases and non-cases, with a 5-year AUC of 0.65(27). The authors suggested that variables such as breast density, alcohol use, and age at primiparity could improve predictions [27]. Furthermore, socioeconomic status might be related to CBC development, as lower socioeconomic status has been associated with higher breast cancer incidence [28], more advanced tumour stage [29,30] and undertreatment [31,32]. Importantly, in the Dutch population these differences were only marginally observed [31,33,34], which was confirmed by nonsignificant contribution of socioeconomic status (based on postal code) in the models predicting LRR and CBC in the present study (data not shown). Moreover, socioeconomic status based on postal code has been shown to be useful in monitoring disparities in healthcare, but it may not be accurate in individual risk prediction [35]. Up to now, there are no models available that more accurately predict CBC than INFLUENCE 3.0 and PREDICTCBC-2.0. Importantly, INFLUENCE 3.0 was not designed for use in women with hereditary breast cancer.

### 4.1 Comparison with INFLUENCE 2.0

The largest advantage of INFLUENCE 3.0 is the inclusion of patients treated with NST. The increasing use of NST over time, mainly in HER2-positive and triple-negative stage II-III breast cancer patients [36,37], largely increases its applicability in clinical practice. Furthermore, INFLUENCE 3.0 included patients with T4 breast cancer, while INFLUENCE 2.0 did not due to a too small sample size. Compared to INFLUENCE 2.0, the following additional factors were examined: menopausal status, presence of DCIS component, results of molecular diagnostics, mode of detection and use of immediate breast reconstruction. The latter two were of added value in the final model. Importantly, the positive association between immediate breast reconstruction and LRR can be explained by the fact that patients with prognostically favourable characteristics more often get an immediate breast reconstruction than patients with prognostically unfavourable characteristics [38]. Menopausal status did not significantly contribute to the current models, probably due to the large predictive value of age. Molecular diagnostics was only performed in ±10 % and 2.5 % of the non-NST and NST population, respectively. This, combined with its likely association with treatment (which was included in the models), might explain its lack of predictive value.

INFLUENCE 3.0 does not predict distant metastasis risk. However, the model was designed to be used as a guidance tool in the shared decision-making process concerning surveillance, which does not aim to detect distant metastases [3]. To assess the risk of distant metastases for patients not treated with NST, however, INFLUENCE 2.0 can still be used.

### 4.2 Clinical implications

After the publication of INFLUENCE 1.0 and 2.0, the model was presented several times at congresses and seminars, and multiple health care professionals expressed their interest in using the model, especially because the generally low LRR risks could be reassuring for patients. Although some health care professionals in the Netherlands have used the model to discuss LRR risks with patients, it was not yet implemented nationwide to support decision-making on the frequency of surveillance visits due to absence of risk estimation of CBC (INFLUENCE 1.0) and risk estimations for patients treated with NST. In addition, a general lack of knowledge on risk communication and effectiveness hampered nationwide implementation. In a multicentre prospective study (SHOUT-BC [39]) on the effectiveness of a patient decision aid including the INFLUENCE 2.0 model (including risk estimations of CBC), significant increases in patient-reported shared decision-making, knowledge on the aim and methods of surveillance and decreases in decisional conflicts and fear of cancer recurrence were observed [40]. Currently, a large prospective study on (cost-)effectiveness of personalised early breast cancer follow-up (NABOR study [41]) is ongoing in which the INFLUENCE 3.0 model is integrated in a decision aid, to support decision-making between patients and caregivers regarding the optimal frequency of surveillance visits for locoregional control. For this purpose, the INFLUENCE 3.0 model will be CE-certified, so it can be used as a medical device in nationwide clinical practice afterwards.”

Consequently, we adapted the sentence on the NABOR study from the conclusion, as we now elaborated on it in the discussion section. In the conclusion it is now stated as follows:

“As part of the NABOR study, it is currently used as a medical device that supports shared decision-making between patients and caregivers regarding the optimal frequency of surveillance visits.

### 4.3 Strengths and limitations

We used optimism-corrected performance measures based on bootstrapping, which is superior to other approaches estimating internal validity [42,43]. Moreover, by looking at quarterly intervals, we could adequately judge the models’ accuracy at different time points during follow-up. This is crucial, as there can be multiple moments during follow-up in which patients and caregivers discuss the frequency of surveillance visits.

The data collection on LRRs has, next to the benefit of a largely reduced workload, some limitations. First, our validation on the data from the first quarter of 2012, showed that we were for ±80 % complete, meaning that we miss ±20 % of all LRRs. Reasons are clinical diagnoses (Palga only contains pathologically confirmed malignancies) or incomplete reporting. This may have resulted in an underestimation of the overall LRR risk and thus misclassification of the outcome variable. A study on the effect of misclassification in logistic regression models showed that AUCs became lower as the degree of misclassification became higher [44]. This implies that AUCs may have been biased and true AUCs would have been higher. We assume a similar impact for survival models. As AUCs in our study still showed moderate performances, we support the use of INFLUENCE in clinical practice.

Data on distant metastases were only registered in case patients were suspected to have a LRR. In case a patient was suspected to have metastases only, the patient file was not searched. Consequently, we missed information on metastases, which may have resulted in inadequate correction for competing risks [9]. Its potential effect was tested by rerunning the INFLUENCE 2.0 models (which did include all information on metastases) on the same cohort as it was developed on, in which we ignored all data on metastases. This was shown not to have clinical relevant effects on the final estimators. This was presumed to be a result of most of the patients with metastases dying not so long after the diagnosis of these metastases (data not yet published).

## 5 Conclusion

INFLUENCE 3.0 moderately predicts LRR and CBC risks up to five years following surgery, in non-metastatic invasive breast cancer patients both treated with and without NST. The models are integrated in an online tool on Evidencio, a platform for medical prediction models (https://www.evidencio.com/models/show/2238).As part of the NABOR study, it is currently used as a medical device that supports shared decision-making between patients and caregivers regarding the optimal frequency of surveillance visits. Notably, it should not be used for treatment decision-making.

## CRediT authorship contribution statement

**M.C. Van Maaren:** Writing – review & editing, Writing – original draft, Visualization, Validation, Software, Resources, Project administration, Methodology, Investigation, Formal analysis, Data curation, Conceptualization. **T.A. Hueting:** Writing – review & editing, Validation, Software, Methodology, Investigation, Formal analysis, Data curation, Conceptualization. **D.J.P. van Uden:** Writing – review & editing. **M. van Hezewijk:** Writing – review & editing. **L. de Munck:** Writing – review & editing. **M.A.M. Mureau:** Writing – review & editing. **P.A. Seegers:** Writing – review & editing. **Q.J.M. Voorham:** Writing – review & editing. **M.K. Schmidt:** Writing – review & editing. **G.S. Sonke:** Writing – review & editing. **C.G.M. Groothuis-Oudshoorn:** Writing – review & editing, Methodology. **S. Siesling:** Writing – review & editing, Resources, Funding acquisition, Conceptualization.

## Data availability

The datasets analysed during the current study are not publicly available due to privacy regulations. However, aggregated data are available from the corresponding author on reasonable request. The dataset can only be made available via the NCR (https://iknl.nl/en/ncr/apply-for-data) after request and approval of a proposal.

## Ethical approval

This study has been approved by the privacy committee of the NCR as well as the NABON-BOOG scientific evaluation committee (reference number K22.075).

## Funding

This study is part of a by 10.13039/501100001826ZonMw funded project (number: 10330032010001). The funding body was not involved in the design of the study and collection, analysis, interpretation of data and in writing the manuscript.

## Declaration of coompeting interest

Tom A. Hueting declares employment at Evidencio. The other authors declare no competing interests.
