# Supplementary material for: The INFLUENCE 3.0 model: Updated predictions of locoregional recurrence and contralateral breast cancer, now also suitable for patients treated with neoadjuvant systemic therapy
Source: Breast. 2024 Oct 28;79:103829. doi: 10.1016/j.breast.2024.103829 (PMC11605451; doi:10.1016/j.breast.2024.103829)
Supplement: Multimedia component 1 [file mmc1.docx]

**Supplementary Table 1: Discrimination results per quarterly time interval**

|  | | **Time (years)** | **Cox** | | | | | | | | | **RSF** | | | | | | | | |
| --- | --- | --- | --- | --- | --- | --- | --- | --- | --- | --- | --- | --- | --- | --- | --- | --- | --- | --- | --- | --- |
|  |  |  | **Original** | | | **Adjusted** | | | **Optimism** | | | **Original** | | | **Adjusted** | | | **Optimism** | | |
|  |  |  | **LB** | **AUC** | **UB** | **LB** | **AUC** | **UB** | **LB** | **mean** | **UB** | **LB** | **AUC** | **UB** | **LB** | **AUC** | **UB** | **LB** | **mean** | **UB** |
| **Non-NST** | **LRR** | 0.25 | 0.85 | **0.94** | 0.99 | 0.91 | **0.93** | 0.95 | -0.061 | **0.013** | 0.047 | 0.98 | **0.99** | 1.00 | 0.94 | **0.97** | 0.99 | 0.039 | **0.021** | 0.008 |
|  |  | 0.50 | 0.90 | **0.93** | 0.97 | 0.92 | **0.93** | 0.94 | -0.022 | **0.002** | 0.031 | 0.96 | **0.97** | 0.98 | 0.94 | **0.95** | 0.96 | 0.023 | **0.022** | 0.025 |
|  |  | 0.75 | 0.88 | **0.92** | 0.94 | 0.91 | **0.92** | 0.93 | -0.031 | **0.004** | 0.019 | 0.94 | **0.95** | 0.97 | 0.92 | **0.94** | 0.94 | 0.020 | **0.015** | 0.029 |
|  |  | 1 | 0.89 | **0.92** | 0.93 | 0.90 | **0.91** | 0.92 | -0.014 | **0.004** | 0.016 | 0.93 | **0.94** | 0.96 | 0.92 | **0.93** | 0.93 | 0.014 | **0.011** | 0.025 |
|  |  | 1.25 | 0.89 | **0.91** | 0.93 | 0.90 | **0.91** | 0.91 | -0.012 | **0.005** | 0.012 | 0.91 | **0.93** | 0.94 | 0.90 | **0.91** | 0.92 | 0.011 | **0.011** | 0.023 |
|  |  | 1.50 | 0.87 | **0.90** | 0.91 | 0.89 | **0.89** | 0.90 | -0.016 | **0.005** | 0.012 | 0.90 | **0.92** | 0.93 | 0.89 | **0.90** | 0.90 | 0.009 | **0.015** | 0.024 |
|  |  | 1.75 | 0.86 | **0.88** | 0.90 | 0.87 | **0.88** | 0.88 | -0.012 | **0.004** | 0.013 | 0.89 | **0.90** | 0.91 | 0.87 | **0.88** | 0.89 | 0.016 | **0.018** | 0.022 |
|  |  | 2 | 0.85 | **0.87** | 0.88 | 0.86 | **0.87** | 0.87 | -0.005 | **0.002** | 0.011 | 0.87 | **0.88** | 0.90 | 0.86 | **0.87** | 0.88 | 0.015 | **0.016** | 0.019 |
|  |  | 2.25 | 0.84 | **0.85** | 0.87 | 0.85 | **0.85** | 0.85 | -0.005 | **0.005** | 0.015 | 0.86 | **0.87** | 0.88 | 0.84 | **0.85** | 0.86 | 0.017 | **0.019** | 0.019 |
|  |  | 2.50 | 0.83 | **0.85** | 0.86 | 0.84 | **0.84** | 0.85 | -0.004 | **0.005** | 0.012 | 0.85 | **0.87** | 0.88 | 0.84 | **0.85** | 0.85 | 0.015 | **0.019** | 0.022 |
|  |  | 2.75 | 0.83 | **0.84** | 0.85 | 0.83 | **0.83** | 0.84 | -0.003 | **0.006** | 0.017 | 0.84 | **0.86** | 0.86 | 0.83 | **0.84** | 0.84 | 0.016 | **0.020** | 0.021 |
|  |  | 3 | 0.82 | **0.83** | 0.85 | 0.82 | **0.83** | 0.83 | -0.004 | **0.006** | 0.017 | 0.84 | **0.85** | 0.86 | 0.82 | **0.83** | 0.84 | 0.016 | **0.020** | 0.026 |
|  |  | 3.25 | 0.81 | **0.82** | 0.83 | 0.81 | **0.81** | 0.81 | -0.001 | **0.008** | 0.019 | 0.82 | **0.84** | 0.85 | 0.81 | **0.81** | 0.82 | 0.019 | **0.022** | 0.029 |
|  |  | 3.50 | 0.80 | **0.81** | 0.83 | 0.80 | **0.80** | 0.81 | -0.001 | **0.008** | 0.021 | 0.82 | **0.83** | 0.84 | 0.80 | **0.81** | 0.81 | 0.019 | **0.024** | 0.031 |
|  |  | 3.75 | 0.80 | **0.81** | 0.82 | 0.80 | **0.80** | 0.80 | -0.001 | **0.008** | 0.022 | 0.81 | **0.82** | 0.84 | 0.79 | **0.80** | 0.81 | 0.018 | **0.023** | 0.033 |
|  |  | 4 | 0.78 | **0.79** | 0.81 | 0.78 | **0.79** | 0.79 | -0.002 | **0.008** | 0.017 | 0.80 | **0.81** | 0.83 | 0.78 | **0.79** | 0.80 | 0.018 | **0.024** | 0.029 |
|  |  | 4.25 | 0.78 | **0.79** | 0.80 | 0.78 | **0.78** | 0.78 | 0.000 | **0.009** | 0.017 | 0.80 | **0.81** | 0.82 | 0.78 | **0.78** | 0.79 | 0.018 | **0.024** | 0.025 |
|  |  | 4.50 | 0.77 | **0.79** | 0.80 | 0.77 | **0.78** | 0.78 | 0.000 | **0.009** | 0.017 | 0.79 | **0.81** | 0.81 | 0.78 | **0.78** | 0.79 | 0.018 | **0.024** | 0.024 |
|  |  | 4.75 | 0.77 | **0.78** | 0.80 | 0.77 | **0.77** | 0.78 | -0.001 | **0.009** | 0.018 | 0.79 | **0.80** | 0.81 | 0.77 | **0.78** | 0.79 | 0.018 | **0.024** | 0.025 |
|  |  | 5 | 0.77 | **0.78** | 0.79 | 0.77 | **0.77** | 0.77 | -0.001 | **0.008** | 0.017 | 0.79 | **0.80** | 0.81 | 0.77 | **0.78** | 0.78 | 0.015 | **0.023** | 0.025 |
|  | **CBC** | 0.25 | 0.63 | **0.84** | 1.00 | 0.50 | **0.82** | 0.93 | 0.126 | **0.021** | 0.069 | 0.99 | **1.00** | 1.00 | 0.50 | **0.91** | 1.00 | 0.492 | **0.085** | 0.002 |
|  |  | 0.50 | 0.62 | **0.76** | 0.85 | 0.71 | **0.75** | 0.80 | -0.091 | **0.007** | 0.053 | 0.91 | **0.94** | 0.97 | 0.83 | **0.87** | 0.92 | 0.086 | **0.070** | 0.049 |
|  |  | 0.75 | 0.62 | **0.73** | 0.80 | 0.68 | **0.72** | 0.75 | -0.057 | **0.013** | 0.050 | 0.90 | **0.92** | 0.94 | 0.81 | **0.83** | 0.87 | 0.089 | **0.081** | 0.070 |
|  |  | 1 | 0.62 | **0.67** | 0.73 | 0.65 | **0.67** | 0.69 | -0.025 | **0.008** | 0.043 | 0.80 | **0.83** | 0.84 | 0.74 | **0.75** | 0.77 | 0.064 | **0.073** | 0.069 |
|  |  | 1.25 | 0.67 | **0.70** | 0.74 | 0.68 | **0.69** | 0.70 | -0.017 | **0.010** | 0.033 | 0.78 | **0.80** | 0.82 | 0.74 | **0.75** | 0.76 | 0.043 | **0.051** | 0.057 |
|  |  | 1.50 | 0.66 | **0.70** | 0.74 | 0.68 | **0.69** | 0.70 | -0.022 | **0.013** | 0.035 | 0.78 | **0.80** | 0.81 | 0.74 | **0.75** | 0.76 | 0.044 | **0.051** | 0.053 |
|  |  | 1.75 | 0.67 | **0.70** | 0.74 | 0.68 | **0.69** | 0.70 | -0.015 | **0.010** | 0.033 | 0.77 | **0.79** | 0.80 | 0.73 | **0.74** | 0.76 | 0.043 | **0.048** | 0.049 |
|  |  | 2 | 0.67 | **0.70** | 0.73 | 0.68 | **0.69** | 0.70 | -0.011 | **0.008** | 0.036 | 0.76 | **0.77** | 0.79 | 0.72 | **0.73** | 0.74 | 0.039 | **0.041** | 0.047 |
|  |  | 2.25 | 0.66 | **0.69** | 0.72 | 0.67 | **0.68** | 0.69 | -0.010 | **0.007** | 0.032 | 0.74 | **0.75** | 0.77 | 0.71 | **0.72** | 0.72 | 0.031 | **0.035** | 0.044 |
|  |  | 2.50 | 0.67 | **0.68** | 0.71 | 0.67 | **0.68** | 0.68 | -0.005 | **0.008** | 0.030 | 0.74 | **0.75** | 0.77 | 0.71 | **0.71** | 0.72 | 0.030 | **0.036** | 0.046 |
|  |  | 2.75 | 0.67 | **0.68** | 0.71 | 0.67 | **0.68** | 0.68 | -0.004 | **0.008** | 0.027 | 0.73 | **0.75** | 0.76 | 0.70 | **0.71** | 0.72 | 0.030 | **0.036** | 0.045 |
|  |  | 3 | 0.66 | **0.68** | 0.70 | 0.66 | **0.67** | 0.68 | -0.003 | **0.008** | 0.023 | 0.73 | **0.74** | 0.75 | 0.69 | **0.70** | 0.71 | 0.031 | **0.035** | 0.044 |
|  |  | 3.25 | 0.65 | **0.67** | 0.69 | 0.66 | **0.66** | 0.67 | -0.005 | **0.006** | 0.022 | 0.72 | **0.73** | 0.74 | 0.69 | **0.70** | 0.70 | 0.029 | **0.033** | 0.042 |
|  |  | 3.50 | 0.65 | **0.67** | 0.69 | 0.66 | **0.66** | 0.67 | -0.005 | **0.007** | 0.024 | 0.71 | **0.73** | 0.74 | 0.69 | **0.69** | 0.70 | 0.027 | **0.033** | 0.042 |
|  |  | 3.75 | 0.65 | **0.66** | 0.68 | 0.65 | **0.66** | 0.66 | -0.005 | **0.008** | 0.024 | 0.71 | **0.72** | 0.74 | 0.68 | **0.69** | 0.69 | 0.030 | **0.035** | 0.043 |
|  |  | 4 | 0.65 | **0.67** | 0.68 | 0.65 | **0.66** | 0.66 | -0.004 | **0.008** | 0.021 | 0.71 | **0.72** | 0.73 | 0.68 | **0.68** | 0.69 | 0.027 | **0.033** | 0.042 |
|  |  | 4.25 | 0.65 | **0.67** | 0.68 | 0.65 | **0.66** | 0.66 | -0.001 | **0.009** | 0.023 | 0.70 | **0.71** | 0.72 | 0.67 | **0.68** | 0.68 | 0.027 | **0.032** | 0.041 |
|  |  | 4.50 | 0.65 | **0.67** | 0.68 | 0.65 | **0.66** | 0.66 | -0.001 | **0.008** | 0.022 | 0.70 | **0.71** | 0.72 | 0.67 | **0.68** | 0.68 | 0.027 | **0.031** | 0.040 |
|  |  | 4.75 | 0.65 | **0.67** | 0.68 | 0.65 | **0.66** | 0.66 | 0.000 | **0.010** | 0.022 | 0.70 | **0.71** | 0.72 | 0.67 | **0.68** | 0.68 | 0.027 | **0.030** | 0.038 |
|  |  | 5 | 0.65 | **0.67** | 0.68 | 0.66 | **0.66** | 0.66 | -0.001 | **0.011** | 0.021 | 0.70 | **0.71** | 0.72 | 0.67 | **0.68** | 0.68 | 0.024 | **0.028** | 0.036 |
| **NST** | **LRR** | 0.25 | 0.85 | **0.89** | 0.95 | 0.87 | **0.89** | 0.90 | -0.021 | **0.000** | 0.048 | 0.95 | **0.97** | 0.99 | 0.87 | **0.93** | 0.96 | 0.081 | **0.034** | 0.022 |
|  |  | 0.50 | 0.84 | **0.89** | 0.93 | 0.87 | **0.89** | 0.90 | -0.032 | **0.000** | 0.033 | 0.91 | **0.94** | 0.96 | 0.90 | **0.91** | 0.93 | 0.012 | **0.023** | 0.037 |
|  |  | 0.75 | 0.84 | **0.89** | 0.92 | 0.87 | **0.88** | 0.89 | -0.033 | **0.006** | 0.028 | 0.91 | **0.93** | 0.95 | 0.90 | **0.91** | 0.92 | 0.012 | **0.019** | 0.031 |
|  |  | 1 | 0.81 | **0.86** | 0.89 | 0.84 | **0.85** | 0.86 | -0.030 | **0.005** | 0.030 | 0.89 | **0.91** | 0.93 | 0.87 | **0.89** | 0.89 | 0.018 | **0.027** | 0.034 |
|  |  | 1.25 | 0.81 | **0.85** | 0.88 | 0.83 | **0.84** | 0.85 | -0.024 | **0.006** | 0.031 | 0.89 | **0.91** | 0.92 | 0.87 | **0.88** | 0.89 | 0.021 | **0.031** | 0.037 |
|  |  | 1.50 | 0.80 | **0.84** | 0.88 | 0.83 | **0.84** | 0.84 | -0.030 | **0.006** | 0.033 | 0.89 | **0.91** | 0.92 | 0.86 | **0.87** | 0.88 | 0.024 | **0.033** | 0.038 |
|  |  | 1.75 | 0.78 | **0.83** | 0.87 | 0.82 | **0.83** | 0.83 | -0.038 | **0.005** | 0.033 | 0.88 | **0.90** | 0.91 | 0.85 | **0.86** | 0.87 | 0.026 | **0.035** | 0.040 |
|  |  | 2 | 0.76 | **0.81** | 0.84 | 0.79 | **0.80** | 0.81 | -0.032 | **0.008** | 0.035 | 0.86 | **0.89** | 0.90 | 0.83 | **0.84** | 0.85 | 0.031 | **0.043** | 0.048 |
|  |  | 2.25 | 0.76 | **0.80** | 0.83 | 0.79 | **0.80** | 0.80 | -0.026 | **0.007** | 0.034 | 0.86 | **0.88** | 0.89 | 0.82 | **0.84** | 0.84 | 0.034 | **0.043** | 0.050 |
|  |  | 2.50 | 0.76 | **0.79** | 0.82 | 0.78 | **0.78** | 0.79 | -0.022 | **0.006** | 0.037 | 0.85 | **0.87** | 0.89 | 0.81 | **0.82** | 0.83 | 0.036 | **0.045** | 0.052 |
|  |  | 2.75 | 0.73 | **0.78** | 0.81 | 0.76 | **0.77** | 0.77 | -0.030 | **0.010** | 0.038 | 0.84 | **0.86** | 0.88 | 0.80 | **0.81** | 0.82 | 0.040 | **0.048** | 0.055 |
|  |  | 3 | 0.73 | **0.77** | 0.81 | 0.76 | **0.76** | 0.77 | -0.028 | **0.009** | 0.038 | 0.84 | **0.86** | 0.87 | 0.80 | **0.81** | 0.82 | 0.042 | **0.049** | 0.056 |
|  |  | 3.25 | 0.73 | **0.77** | 0.80 | 0.75 | **0.76** | 0.76 | -0.026 | **0.009** | 0.037 | 0.83 | **0.85** | 0.87 | 0.79 | **0.80** | 0.81 | 0.041 | **0.050** | 0.059 |
|  |  | 3.50 | 0.72 | **0.76** | 0.79 | 0.74 | **0.75** | 0.75 | -0.026 | **0.010** | 0.035 | 0.83 | **0.84** | 0.86 | 0.78 | **0.79** | 0.80 | 0.045 | **0.052** | 0.060 |
|  |  | 3.75 | 0.72 | **0.75** | 0.78 | 0.74 | **0.74** | 0.75 | -0.021 | **0.009** | 0.034 | 0.82 | **0.84** | 0.86 | 0.78 | **0.79** | 0.80 | 0.044 | **0.052** | 0.063 |
|  |  | 4 | 0.72 | **0.75** | 0.78 | 0.74 | **0.74** | 0.75 | -0.018 | **0.010** | 0.034 | 0.82 | **0.84** | 0.86 | 0.78 | **0.79** | 0.80 | 0.042 | **0.051** | 0.061 |
|  |  | 4.25 | 0.72 | **0.75** | 0.78 | 0.73 | **0.74** | 0.74 | -0.017 | **0.009** | 0.034 | 0.82 | **0.84** | 0.86 | 0.77 | **0.78** | 0.79 | 0.045 | **0.053** | 0.063 |
|  |  | 4.50 | 0.71 | **0.74** | 0.77 | 0.73 | **0.73** | 0.74 | -0.016 | **0.010** | 0.036 | 0.81 | **0.83** | 0.85 | 0.77 | **0.78** | 0.79 | 0.045 | **0.053** | 0.064 |
|  |  | 4.75 | 0.72 | **0.74** | 0.78 | 0.73 | **0.74** | 0.74 | -0.014 | **0.009** | 0.036 | 0.82 | **0.83** | 0.85 | 0.77 | **0.78** | 0.79 | 0.045 | **0.052** | 0.062 |
|  |  | 5 | 0.71 | **0.74** | 0.77 | 0.72 | **0.73** | 0.73 | -0.013 | **0.009** | 0.038 | 0.81 | **0.83** | 0.84 | 0.76 | **0.77** | 0.78 | 0.044 | **0.053** | 0.063 |
|  | **CBC** | 0.25 | 0.50 | **0.64** | 0.76 | 0.44 | **0.60** | 0.66 | 0.060 | **0.040** | 0.102 | 0.91 | **0.93** | 0.95 | 0.63 | **0.78** | 0.85 | 0.272 | **0.156** | 0.098 |
|  |  | 0.50 | 0.54 | **0.66** | 0.78 | 0.49 | **0.63** | 0.68 | 0.057 | **0.028** | 0.097 | 0.90 | **0.93** | 0.95 | 0.68 | **0.79** | 0.86 | 0.223 | **0.143** | 0.092 |
|  |  | 0.75 | 0.57 | **0.67** | 0.77 | 0.53 | **0.63** | 0.70 | 0.035 | **0.047** | 0.061 | 0.90 | **0.92** | 0.94 | 0.72 | **0.80** | 0.86 | 0.179 | **0.125** | 0.084 |
|  |  | 1 | 0.56 | **0.65** | 0.76 | 0.54 | **0.62** | 0.68 | 0.022 | **0.037** | 0.082 | 0.89 | **0.91** | 0.94 | 0.73 | **0.77** | 0.83 | 0.159 | **0.144** | 0.111 |
|  |  | 1.25 | 0.57 | **0.65** | 0.75 | 0.54 | **0.61** | 0.69 | 0.023 | **0.040** | 0.066 | 0.87 | **0.91** | 0.95 | 0.72 | **0.77** | 0.81 | 0.152 | **0.141** | 0.142 |
|  |  | 1.50 | 0.57 | **0.65** | 0.73 | 0.53 | **0.61** | 0.66 | 0.044 | **0.046** | 0.064 | 0.87 | **0.90** | 0.95 | 0.71 | **0.75** | 0.79 | 0.152 | **0.150** | 0.156 |
|  |  | 1.75 | 0.58 | **0.67** | 0.74 | 0.53 | **0.63** | 0.68 | 0.054 | **0.046** | 0.064 | 0.87 | **0.90** | 0.93 | 0.73 | **0.76** | 0.80 | 0.138 | **0.134** | 0.129 |
|  |  | 2 | 0.61 | **0.67** | 0.73 | 0.55 | **0.63** | 0.68 | 0.063 | **0.036** | 0.057 | 0.86 | **0.89** | 0.92 | 0.73 | **0.76** | 0.79 | 0.131 | **0.127** | 0.121 |
|  |  | 2.25 | 0.60 | **0.66** | 0.73 | 0.52 | **0.61** | 0.65 | 0.074 | **0.041** | 0.073 | 0.85 | **0.88** | 0.91 | 0.72 | **0.76** | 0.79 | 0.130 | **0.123** | 0.120 |
|  |  | 2.50 | 0.60 | **0.66** | 0.72 | 0.52 | **0.62** | 0.65 | 0.079 | **0.044** | 0.072 | 0.85 | **0.88** | 0.91 | 0.71 | **0.76** | 0.78 | 0.135 | **0.125** | 0.124 |
|  |  | 2.75 | 0.60 | **0.63** | 0.71 | 0.53 | **0.60** | 0.64 | 0.065 | **0.032** | 0.071 | 0.84 | **0.87** | 0.90 | 0.71 | **0.75** | 0.78 | 0.133 | **0.121** | 0.119 |
|  |  | 3 | 0.61 | **0.63** | 0.71 | 0.53 | **0.60** | 0.64 | 0.072 | **0.031** | 0.070 | 0.85 | **0.87** | 0.90 | 0.71 | **0.76** | 0.78 | 0.135 | **0.118** | 0.118 |
|  |  | 3.25 | 0.61 | **0.64** | 0.71 | 0.55 | **0.61** | 0.64 | 0.061 | **0.036** | 0.061 | 0.84 | **0.87** | 0.90 | 0.72 | **0.76** | 0.78 | 0.127 | **0.116** | 0.120 |
|  |  | 3.50 | 0.61 | **0.65** | 0.70 | 0.55 | **0.61** | 0.64 | 0.064 | **0.041** | 0.058 | 0.84 | **0.87** | 0.90 | 0.72 | **0.76** | 0.78 | 0.113 | **0.113** | 0.121 |
|  |  | 3.75 | 0.61 | **0.65** | 0.70 | 0.56 | **0.61** | 0.64 | 0.054 | **0.039** | 0.059 | 0.83 | **0.86** | 0.90 | 0.72 | **0.75** | 0.78 | 0.112 | **0.115** | 0.118 |
|  |  | 4 | 0.61 | **0.65** | 0.69 | 0.55 | **0.61** | 0.63 | 0.055 | **0.041** | 0.062 | 0.83 | **0.86** | 0.88 | 0.71 | **0.74** | 0.77 | 0.117 | **0.119** | 0.106 |
|  |  | 4.25 | 0.61 | **0.65** | 0.69 | 0.55 | **0.61** | 0.62 | 0.061 | **0.041** | 0.067 | 0.82 | **0.86** | 0.88 | 0.70 | **0.73** | 0.76 | 0.114 | **0.122** | 0.114 |
|  |  | 4.50 | 0.61 | **0.65** | 0.69 | 0.55 | **0.61** | 0.62 | 0.061 | **0.041** | 0.067 | 0.82 | **0.85** | 0.88 | 0.70 | **0.73** | 0.76 | 0.121 | **0.121** | 0.117 |
|  |  | 4.75 | 0.61 | **0.65** | 0.69 | 0.54 | **0.60** | 0.62 | 0.065 | **0.043** | 0.068 | 0.82 | **0.85** | 0.88 | 0.70 | **0.73** | 0.76 | 0.121 | **0.119** | 0.118 |
|  |  | 5 | 0.61 | **0.64** | 0.69 | 0.55 | **0.60** | 0.61 | 0.060 | **0.044** | 0.080 | 0.81 | **0.84** | 0.87 | 0.69 | **0.73** | 0.76 | 0.123 | **0.114** | 0.107 |

Abbreviations: LB = 95% lower bound. AUC = area under the receiver operating characteristic curve. UB = 95% upper bound. Mean values are depicted in bold. Values per year are shaded.
