# Supplementary material for: The INFLUENCE 3.0 model: Updated predictions of locoregional recurrence and contralateral breast cancer, now also suitable for patients treated with neoadjuvant systemic therapy
Source: Breast. 2024 Oct 28;79:103829. doi: 10.1016/j.breast.2024.103829 (PMC11605451; doi:10.1016/j.breast.2024.103829)
Supplement: Multimedia component 2 [file mmc2.docx]

**Supplementary Table 2. Mean hazard ratios and 95% confidence intervals of 100 bootstrap samples on which the Cox regression model predicting LRR in the non-NST cohort was developed.**

|  |  | **HR** | **95% LCL** | **95% UCL** |
| --- | --- | --- | --- | --- |
| Age | Age knot 1 | 1 |  |  |
|  | Age knot 2 | 1.00 | 0.98 | 1.01 |
|  | Age knot 3 | 1.00 | 0.98 | 1.01 |
| pT stage | pT1 | 1 |  |  |
|  | pT2 | 1.41 | 1.22 | 1.61 |
|  | pT3 | 1.45 | 0.93 | 2.11 |
|  | pT4 | 1.31 | 0.61 | 2.31 |
| pN stage | pN0 | 1.00 |  |  |
|  | pN1 | 1.58 | 1.35 | 1.88 |
|  | pN2 | 2.35 | 1.59 | 3.48 |
|  | pN3 | 3.45 | 2.58 | 4.80 |
| Sublocalisation | Outer quadrants | 1 |  |  |
|  | Inner quadrants | 1.34 | 1.14 | 1.55 |
|  | Central parts | 0.96 | 0.73 | 1.26 |
|  | Overlapping lesions | 1.15 | 0.99 | 1.35 |
| Histological tumour type | Ductal | 1 |  |  |
|  | Lobular | 0.78 | 0.63 | 0.99 |
|  | Mixed ductal and lobular | 0.86 | 0.55 | 1.35 |
|  | Other | 0.89 | 0.65 | 1.20 |
| Differentiation grade | Grade 1 | 1 |  |  |
|  | Grade 2 | 1.68 | 1.32 | 2.09 |
|  | Grade 3/4 | 2.68 | 2.19 | 3.32 |
| HR status ± endocrine therapy | Positive + treatment | 1 |  |  |
|  | Positive - treatment | 2.27 | 1.94 | 2.78 |
|  | Negative | 3.00 | 2.48 | 3.59 |
| HER2 status ± targeted therapy | Negative | 1 |  |  |
|  | Positive + treatment | 0.30 | 0.18 | 0.41 |
|  | Positive - treatment | 1.17 | 0.92 | 1.50 |
| Mode of detection | Symptoms | 1 |  |  |
|  | Screening | 0.60 | 0.52 | 0.73 |
| Surgery ± RT ± immediate breast reconstruction | BCS + RT | 1 |  |  |
|  | BCS - RT | 5.09 | 3.86 | 6.82 |
|  | mastectomy + RT – immediate breast reconstruction | 0.94 | 0.67 | 1.23 |
|  | mastectomy + RT + immediate breast reconstruction | 1.16 | 0.52 | 2.10 |
|  | mastectomy – RT – immediate breast reconstruction | 2.60 | 2.18 | 2.99 |
|  | mastectomy – RT + immediate breast reconstruction | 3.46 | 2.58 | 4.43 |
| Chemotherapy | No | 1 |  |  |
|  | Yes | 0.61 | 0.50 | 0.72 |

* As age is modeled using restricted cubic splines, the interpretation of the coefficients is not straightforward, as the effect of age on risk of LRR is a function of multiple regression coefficients. Abbreviations: LRR = locoregional recurrence, NST = neoadjuvant systemic treatment, HR = hazard ratio, LCL = lower confidence limit, UCL = upper confidence limit, pT = pathological T stage, pN = pathological nodal stage, HER2 = human epidermal growth factor 2, RT = radiation therapy.
