# Supplementary material for: The INFLUENCE 3.0 model: Updated predictions of locoregional recurrence and contralateral breast cancer, now also suitable for patients treated with neoadjuvant systemic therapy
Source: Breast. 2024 Oct 28;79:103829. doi: 10.1016/j.breast.2024.103829 (PMC11605451; doi:10.1016/j.breast.2024.103829)
Supplement: Multimedia component 3 [file mmc3.docx]

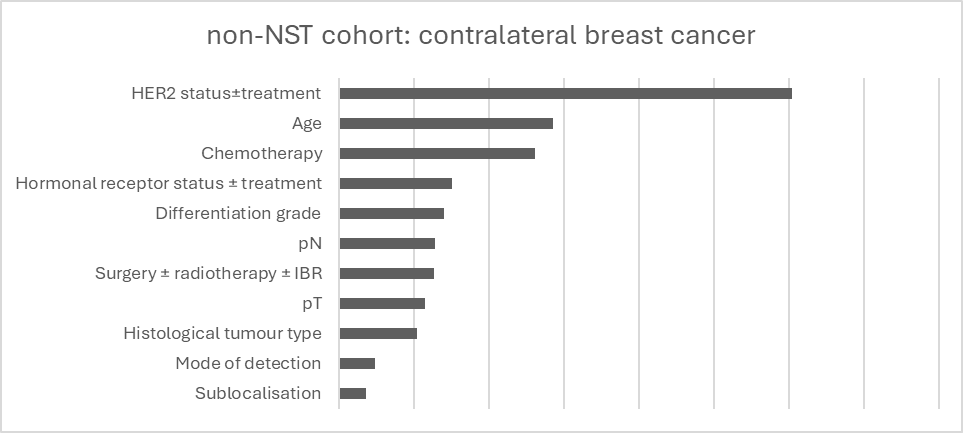

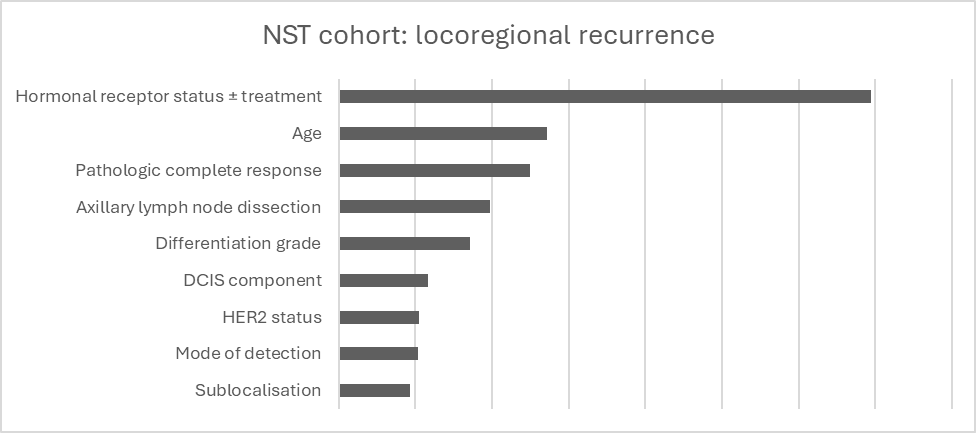

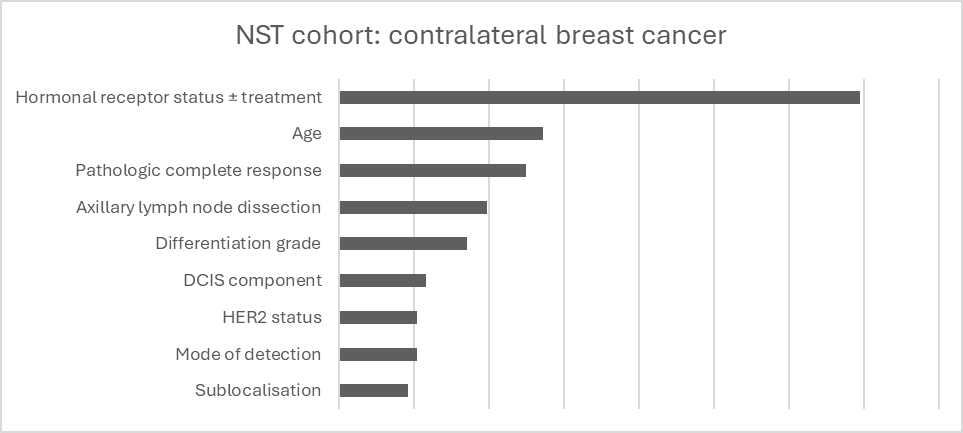


**Supplementary Figure 1. Importance ranking of variables in the random survival forest models**

All variables are scaled and in this way, the variables can be compared with each other (i.e. the variable HER2 status±treatment is more than twice as important in the model as the variable age, in the upper panel) Due to the black-box approach of the random survival forest model, absolute variable importance could not be obtained. Abbreviations: IBR = immediate breast reconstruction, pN = pathological nodal classification, pT = pathological tumour classification, NST = neoadjuvant systemic treatment.
